# Supplementary material for: Verhulst-type equation and the universal pattern for global population growth
Source: PLoS One. 2025 May 19;20(5):e0323165. doi: 10.1371/journal.pone.0323165 (PMC12088597; doi:10.1371/journal.pone.0323165)
Supplement: S1 Appendix — [128–135], and subsequently their numerical filtering. (DOCX) [file pone.0323165.s001.docx]

**S1 Appendix:**

**Global population data applied in the given report and in ref. [97], based on numerical filtering of data from a few reference sources**

| Year | Population  (milion) | Year | Population  (milion) | Year | Population  (milion) | Year | Population  (milion) |
| --- | --- | --- | --- | --- | --- | --- | --- |
| -10000 | 2 | 100 | 195 | 1790 | 920 | 1913 | 1793 |
| -8500 | 2.2 | 200 | 202 | 1800 | 990 | 1914 | 1812 |
| -8000 | 2.5 | 250 | 203 | 1802 | 1015 | 1916 | 1837 |
| -7000 | 2.7 | 300 | 205 | 1805 | 1035 | 1918 | 1832 |
| -6000 | 3.1 | 360 | 200 | 1810 | 1050 | 1920 | 1852 |
| -5000 | 4.2 | 380 | 199 | 1815 | 1055 | 1922 | 1887 |
| -4500 | 5.5 | 410 | 197 | 1820 | 1080 | 1924 | 1921 |
| -4000 | 6.7 | 450 | 208 | 1822 | 1094 | 1925 | 2000 |
| -3500 | 9 | 500 | 210 | 1824 | 1109 | 1926 | 1972 |
| -3000 | 13 | 550 | 205 | 1828 | 1144 | 1928 | 2021 |
| -10000 | 2 | 600 | 210 | 1832 | 1154 | 1930 | 2090 |
| -8500 | 2.2 | 650 | 215 | 1835 | 1164 | 1932 | 2116 |
| -8000 | 2.5 | 700 | 220 | 1838 | 1184 | 1934 | 2175 |
| -7000 | 2.7 | 750 | 225 | 1841 | 1203 | 1937 | 2224.5 |
| -6000 | 3.1 | 800 | 230 | 1844 | 1218 | 1940 | 2313 |
| -2500 | 19 | 890 | 240 | 1847 | 1243 | 1945 | 2417 |
| -2000 | 27 | 950 | 260 | 1850 | 1268 | 1947 | 2449 |
| -1500 | 37 | 1000 | 280 | 1854 | 1278 | 1948 | 2493 |
| -1250 | 46 | 1050 | 320 | 1857 | 1292 | 1950 | 2526 |
| -1000 | 54 | 1100 | 353 | 1860 | 1297 | 1951 | 2543.1 |
| -10000 | 2 | 1150 | 370 | 1863 | 1287 | 1952 | 2590.3 |
| -8500 | 2.2 | 1200 | 395 | 1866 | 1278 | 1953 | 2640.3 |
| -8000 | 2.5 | 1250 | 409 | 1869 | 1298 | 1954 | 2691.9 |
| -7000 | 2.7 | 1300 | 415 | 1870 | 1276 | 1955 | 2746.07 |
| -6000 | 3.1 | 1330 | 425 | 1873 | 1327 | 1956 | 2801 |
| -5000 | 4.2 | 1360 | 464 | 1875 | 1325 | 1957 | 2857.87 |
| -4500 | 5.5 | 1380 | 385 | 1876 | 1362 | 1958 | 2916.11 |
| -4000 | 6.7 | 1425 | 360 | 1880 | 1381 | 1959 | 2970.29 |
| -3500 | 9 | 1450 | 380 | 1884 | 1421 | 1960 | 3019.233 |
| -3000 | 13 | 1475 | 410 | 1888 | 1485 | 1961 | 3068.37 |
| -2500 | 19 | 1500 | 457 | 1890 | 1506 | 1962 | 3126.69 |
| -2000 | 27 | 1540 | 465 | 1892 | 1525 | 1963 | 3195.78 |
| -1500 | 37 | 1575 | 530 | 1894 | 1545 | 1964 | 3267.21 |
| -1250 | 46 | 1600 | 544 | 1896 | 1570 | 1965 | 3337.11 |
| -1000 | 54 | 1620 | 556 | 1898 | 1585 | 1966 | 3406.42 |
| -750 | 61 | 1660 | 590 | 1900 | 1654 | 1967 | 3475.45 |
| -650 | 70 | 1700 | 603 | 1902 | 1639 | 1968 | 3546.81 |
| -500 | 100 | 1720 | 608 | 1904 | 1669 | 1969 | 3620.66 |
| -400 | 140 | 1735 | 656 | 1906 | 1703 | 1970 | 3667 |
| -200 | 149 | 1750 | 692 | 1908 | 1728 | 1971 | 3770.16 |
| -10 | 175 | 1760 | 760 | 1910 | 1777 | 1972 | 3844.8 |
| 1 | 188 | 1770 | 820 | 1912 | 1788 | 1973 | 3920.25 |

| Year | Population  (milion) | Year | Population  (milion) | Year | Population  (milion) |
| --- | --- | --- | --- | --- | --- |
| 1974 | 3995.52 | 1991 | 5406.25 | 2008 | 6811.6 |
| 1975 | 4069.43 | 1992 | 5492.69 | 2009 | 6898.31 |
| 1976 | 4142.51 | 1993 | 5577.43 | 2010 | 6985.6 |
| 1977 | 4215.77 | 1994 | 5660.73 | 2011 | 7073.13 |
| 1978 | 4289.66 | 1995 | 5743.22 | 2012 | 7161.7 |
| 1979 | 4365.58 | 1996 | 5812 | 2013 | 7250.59 |
| 1980 | 4444.01 | 1997 | 5906.48 | 2014 | 7318 |
| 1981 | 4524.63 | 1998 | 5980 | 2015 | 7426.6 |
| 1982 | 4607.98 | 1999 | 6062 | 2016 | 7492 |
| 1983 | 4691.88 | 2000 | 6148.9 | 2017 | 7599.82 |
| 1984 | 4775.84 | 2001 | 6230.75 | 2018 | 7683.79 |
| 1985 | 4861.73 | 2002 | 6312.41 | 2019 | 7743 |
| 1986 | 4950.06 | 2003 | 6393.9 | 2020 | 7840.95 |
| 1987 | 5040.98 | 2004 | 6471 | 2021 | 7909.3 |
| 1988 | 5132.29 | 2005 | 6558.18 | 2022 | 8000 |
| 1989 | 5223.7 | 2006 | 6641.42 | 2023 | 8045 |
| 1990 | 5316.18 | 2007 | 6717 |  |  |
