# Supplementary material for: Verhulst-type equation and the universal pattern for global population growth
Source: PLoS One. 2025 May 19;20(5):e0323165. doi: 10.1371/journal.pone.0323165 (PMC12088597; doi:10.1371/journal.pone.0323165)
Supplement: Attachment — Submitted filename: Review Plos.pdf [file pone.0323165.s002.pdf]

The paper entitled “Verhulst Equation… Population Grouth” has studied the global population since Antropocene period up to day. The core of the work is very interesting and useful for the global prospects. Even though the work is in the right direction, there are the following points to be amended:

1. It is not clear the contribution of this paper to this topic. what is the new from this work. This have to be presented in the abstract or at the end of introduction.
2. The part of theory is too big. It is preferable to give the necessary equations, only.
3. It is referred the phrase “critical system” which it seems to be similar with “critical phenomena” in Physics, in Economy etc. Authors have to examine possible similarities excluding more information.
4. Authors analyze the increase of Global Population using the Theory of Minimum Least Squares (TMLS) . According this theory the parameters of **a** and **b** can give additional information.
5. The data of Global Population are sufficient in order to be applied Arma and Arima models, Neural Nets or Chaos Theory for prediction reasons. Also from the time series analysis can be found the entropy of the system.

The paper needs major revision.
