# Supplementary material for: Verhulst-type equation and the universal pattern for global population growth
Source: PLoS One. 2025 May 19;20(5):e0323165. doi: 10.1371/journal.pone.0323165 (PMC12088597; doi:10.1371/journal.pone.0323165)
Supplement: Attachment — Submitted filename: Response letter vwith Lehman comment.pdf [file pone.0323165.s005.pdf]

Dear Editor,

Attached please find the revised manuscript ‘ *Verhulst Equation and the Universal Pattern for Global Population Growth’, by Agata Angelika Sojecka and Aleksandra Drozd-Rzoska* in which all reasonable comments of reviewers have been positively addressed.

Note general supplementations:

1. The paper has been deeply cleaned regarding language.
2. Following reviewers comments the new Figure 4 + related comments at the end of Results & Discussion section
3. Note ca. 40 new references, added to meet reviewers' requirements
4. Note the strongly corrected Abstract and Conclusions, to meet reviewers expectations.
5. Note the report [97] ‘Sojecka, AA, Drozd-Rzoska A. *Global population: from Super-Malthus behavior to Doomsday criticality*. **Scientific Reports**. 2024; 14; 9853. https://doi.org/10.1038/s41598-024-60589-3 - which is strictly related to the given paper. It is a complementary paper submitted to Nature-Springer a month later (5th March 2024) after the submission to PlosOne and very quickly accepted after 3 professional and positive opinions, so it appeared in May 2024.

   The results from this report are significant for the given report. This report is recalled a few times, since it significantly support the results presented in the given manuscript.
6. Note that the re-submitted report is ‘**placed’ in PlosOne template** (available in the Net), and follows the template's requirements. All references are corrected according to PlosOne rules.

Next general issue: ‘*Please remove any funding-related text from the manuscript’*/

Response: : The report was supported by the grant of ‘"*National Center for Science (NCN, Poland), grant ref. 2022/45/B/ST5/04" headed by S.J. Rzoska. The funders had no role in study design, data collection and analysis, decision to publish, or preparation of the manuscript’.*

Note that for the given type of grants usually funders does not influence such decisions, essential is only the significance to the Project. Following the send requirement the Acknowledgent with funding statement has been removed.

Nevertheless it has to be presented in the finally accepted text. Without the statement any fee payment will be not possible.

Next issue general : ‘ *Can you please comment on this work's relationship with the Lehman paper, in the cover letter or an "Other" file? Do you consider your work to contradict or dispute any of the findings in the Lehman paper? If so, we may invite a signed review from the authors of the Lehman paper. If findings only build on this or extend their work, we would be grateful if you can confirm this.’*

Response: One aspect of the Report of Lehman et al., was a basic inspiration – namely, the indication of the per capita relative population growth rate G_N and finding its very characteristic changes via two lines with opposite slopes: from 10 000 BCE till ~1965 and from 1965. It is shown in Fig. 3 of the ref. by Lehman et al.

In the given paper, we obtained the same behavior as for the new set of data: see Figure 2. But the more focused analysis shown in Figure 3 revealed that the first linear domain is much shorter: it starts from 1 000 CE. Much shorter but still long.

More important is the analysis – in fact, very simple and possible for a ‘student’ with a bit of experience in mathematics – that such linear behavior determines the optimal pair of

parameters for the Verhulst dependence in each mentioned should describe population rise for within the time- and population scale determined by the linear domain. But it does not happen. Hence, purely functional analysis of the Verhulst equation in the frame of the finding/discovery shown in Figure 3 Lehman et al.) and in Figures 2 and 3 of the given paper proves that using a sequence of n-pair (r,s) parameters is not allowed. It question of course some results of the report by Lehman et al, but much more very classic report by Volterra and earlier by Pearl.
So – does this report contradict the report by Lehman et al : YES and NO. The last means great credit for showing 'universal' G_N changes, although Lehman et al. did not draw any conclusions from this finding, leaving the 'cognitive space' for others.

.

The Academic Editor (AE) suggested:

1. **AE:** *Clarify the paper's contribution to the topic and highlight what is new in the abstract or introduction. Increase references in the introduction to better engage with existing literature. Include pictorial illustrations to summarize the story and improve the article.*
   Response: The Introduction has been cleaned and clarified, and the re-written, precise motivation is no given. It is strongly stressed that the report is related to the Verhulst equation portrayal and test of the coupled hypothetical universality of the per-capita relative population growth coefficient. The novelty related to the way of global population data and the distortions – sensitive analysis. In the Introduction there are numerous new references, particularly from 2023-2024, to show the current significance of the topic. Note the new Figure 4 and correction in Figures 2 and 3.
2. **AE:** *The theoretical section needs to be shorter and include only essential equations. In the introduction, clearly define the motivation, research gap, and research question.*
   Response: the reduction of the number of equations does not agree with suggestions of reviewers and could make the paper not-clear. Shortening the Introduction is in disagreement with the suggestion of adding significantly more references and report clarification.
   The final part of results and discussion has been re-written, and now cognitive challenge/gap and motivation/research questions… are explicitly and precisely presented. See lines 94 – 117**.**
3. **AE:** *Examine similarities between "critical system" and "critical phenomena" in other fields.*
   Responce: the explicit link of these names to Critical Phenomena Physics, a commonly known branch of physics and complex systems science, has been recalled a few times. Note particularly the end of the Results and Discussion section.
4. **AE:** *Use the Theory of Minimum Least Squares (TMLS) to provide additional information on parameters a and b.*
   Response: First note that the Savitzky – Golay filtering routine is better explained in the Methods section, with supporting references. I guess this comment is related to parameters given in Table I, which was determined to use the linear regression routine.

It is so standard and popular procedure that its explanation has minimal sense, in my opinion. It is now explicitly stated in the caption of Table I.

5. **AE:** *Apply ARMA, ARIMA models, Neural Networks, or Chaos Theory for prediction purposes, and analyze the system's entropy from the time series data. Improve the theoretical background and explain the study's relevance and contribution to existing knowledge*

Response: Note that the theoretical background is well explained now. It follows a new path of global population analysis. It has been introduced, for the first time, in the report mentioned above (in the reference list, it is position 97). In fact, this report in PlosOne could be the first communicate on this cognitive path, but we waited 6 months (!) for opinions (!)

As for ARMA, ARIMA etc… models – this issue is explained in commented at the end Conclusions section. Lines 536-563.

As the profesionalist in complex systems/matter science, I should additionally comment this issue. This suggestion is well beyond the target and motivation of the given report. Second, so fat there are no reasonably valueable report applying such approaches for global population. This comment is related to action with real global population data. There are pure model-theoretical analyses, but they are meaningless without implementation for real data. This lack is not accidental.

Third, the entropy analysis for the time-related non-monotonic global population data is strange. I am not surprised that it has not been done before.

<u>This summary of AE comments and Authors explanations address all issues indicated by Reviewers</u>.

In fact, it was difficult to address all these comment, because their targets were contradictory. It was suggested that the report should be shorter, with simple and straightforward targets and motivations

On the other hand, some specific suggestions were well beyond the current state-of-the-art, and could strongly defocus the report.

Nevertheless, I made the best to meet all reasonable suggestions.

I submitted the basic report a lot of months ago. So now, I will be grateful for the fast final decision: Yes or No.

Thanking for Your troubles
Yours Sincerely

Prof. Aleksandra Drozd-Rzoska
Institute of High Pressure Physics
Polish Academy of Sciences
