# Supplementary material for: Verhulst-type equation and the universal pattern for global population growth
Source: PLoS One. 2025 May 19;20(5):e0323165. doi: 10.1371/journal.pone.0323165 (PMC12088597; doi:10.1371/journal.pone.0323165)
Supplement: Attachment — Submitted filename: Review-GlobalPopulation-2024.pdf [file pone.0323165.s004.pdf]

UNIVERSITY OF MINNESOTA

Twin Cities Campus

Department of Ecology, Evolution and Behavior

College of Biological Sciences

100 Ecology Building
1987 Upper Buford Circle
St. Paul, MN 55108-6097

Office: 612-625-5700
Fax: 612-624-6777
http://www.cbs.umn.edu/eeb

MEMO TO: Agata Angelika Sojecka and Aleksandra Drozd-Rzoska
FROM: Clarence Lehman, also on behalf of co-authors Shelby Loberg and Michael Wilson
SUBJECT: Thoughts on "Verhulst Equation and the Universal Pattern for Global Population Growth"
DATE: October 18, 2024

I and my co-authors are pleased to learn that our 2021 paper provided inspiration for you to look at the subject with a different method. The editors of PLOS ONE asked if I would be willing to write a signed review letter concerning your manuscript, which I am happy to do.

It seems that the two papers reach similar conclusions, using different methods. Our approach was to apply three-dimensional differential equations representing the unified models of ecology, which can collapse to a single-dimensional equation under certain conditions, such as strong mutualism. This I would characterize as an inverse scientific modeling approach. Your approach for smoothing and fitting the data would be characterized as a statistical or computational approach. Is that how you would characterize it?

1. Comparison of the data

Your data are from different sources than ours, yet appear to be rather consistent with ours. To see the similarities graphically, I took our Figure 3 and converted it to a semilog scale on the vertical access and a linear scale on the horizontal axis, to match your Figure 1. Here are the results:

Your Figure 1

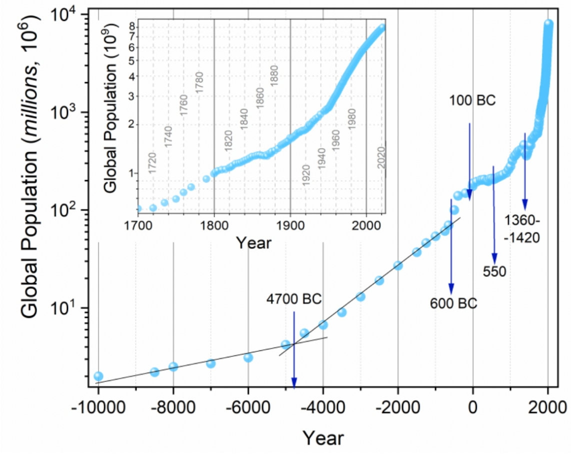

Our Figure 3, scaled to match

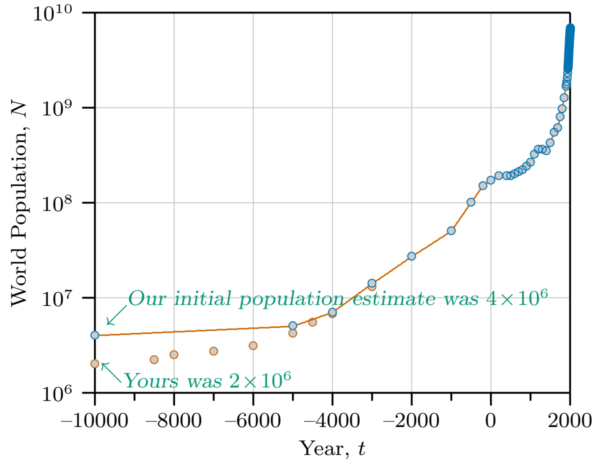

Our data on the right are shown with blue dots and a red line connecting them. Your data, up to the year minus 3000, are shown on the right with red dots and no line connecting them. A main difference is that our population estimate was 4 million at the beginning and yours is only half that, 2 million. My feeling is that either value could be plausible—it is very hard to know the human population of the earth accurately twelve-thousand years ago.

Both of these curves, yours and ours, show a notable upward curvature—for example if one would fit a strictly quadratic curve to the data. On a semilog plot like this, an upward curvature indicates faster than exponential growth, such as what we have named orthologistic growth, a straight line indicates exponential growth, and a downward curvature represents slower than exponential growth, such as logistic growth explained by Verhulst. In any case, both sets of data support the same conclusion—that human population growth was faster than exponential, with various ups and downs, through this phase.

2. Comparison of the methods

Our approach was to take the unified models of ecology, as explained in our 2020 paper (BioScience 70:415–426), and apply them to various stages of human population growth. In the period starting about 12,000 years ago, our species developed mass mutualisms with plants and animals becoming domesticated, leading to a strong upward trend in per capita population growth, with noticeable deviations due to multiple wars, diseases, and other events throughout this time. You find the same upward trend by smoothing and fitting the data.

How to interpret each approach? We take it as the natural ecological consequence of strong mutualisms developed during the agricultural phase. You are applying, it looks to me, a modified form of the Verhulst–Pearl–Reed equation, with a negative carrying capacity to induce the observed dynamics. I'm not sure that is what Pearl and other demographers did, however. Didn't they just apply repeatedly larger and larger carrying capacities and growth rates as human populations repeatedly outstripped previous estimates? Did they ever consider what a negative carrying capacity might mean? Indeed, what does a negative carrying capacity mean, logically, intuitively?

In any case, with very different approaches, we both seem to reach the same conclusion: That human populations grew faster than exponentially throughout the developing agricultural period, with various ups and downs, but sometime in the mid-twentieth century, it shifted to classical logistic growth, appearing to head toward an equilibrium. We also point out, based on Figure 4 drawn by our late co-author Eville Gorham, that global population could actually decline in the not too distant future.

The editors were thinking your manuscript might be a rebuttal to our paper, and on first reading it seemed to me that way too, but on careful thought, it seems to be a confirmation. How do you see it?

3. Some suggestions

On reading the manuscript, we noticed some things that could/should be improved, and I can list those below.

In the abstract, it says "the Verhulst relation with such effective parameters does not describe $P(t)$ changes." I don't understand what that means. Isn't it all about $P(t)$?

Earlier in the abstract, you write "$G_P(P) = \big(dP(t)/P(t)\big)/dt = d\ln P(t)/dt$." I just wanted to point out that after many years of explaining these equations to thousands of students, I found that writing it as $\big(1/P(t)\big)\,\big(dP(t)/dt\big)$ makes it easier to understand, because one can focus successively on the dP as 'the change in population,' the dt as 'per unit of time', and the $1/P$ as 'per individual in the population.' Also, though it is true that it is equivalent to the natural-log formulation, that formulation will add further confusion to all but those experienced in mathematics. And many interested readers of the paper will not be in that group. (One final little thing—in your formula, you have the 'ln' for natural logarithm italicized, whereas typical mathematical style would be unitalicized for that. I'm pointing it out because copy editors don't always catch such things in equations.)

In the introduction—at least in what I think is the most recent version of your manuscript—it starts out, "The Anthropocene epoch began 10,000 years ago ..." Don't you mean 12,000 years ago? An easy mistake to make I know, because I did the exact same thing communicating with my co-authors just yesterday! Also, since there are differing views, you might want to say something like "The Anthropocene epoch, as we consider it here, began 12,000 years ago ..." Geologists and others don't agree on this term or when it should begin, or even if it is justified to name this epoch. But if you add the "as we consider it here" phrase, you should be okay. Also, the "due to global warming" could be controversial. We, for example, tie it to the massive global ecological changes due to developing agriculture and civilization. There were numerous other global-warming times without faster-than-exponential population growth, tied to the Milankovitch cycles, for example.

Also in the introduction, I flagged the word "egoistic" as perhaps not the right word for this thought in a scientific paper. I saw similar things elsewhere, and perhaps it would be good for you to find an additional copy editor whose native language is English to look for and review such things.

Later in the introduction, something is quite wrong with the factoring in Equation 3. In my copy of the manuscript it says,

$$\frac{dP(t)}{dt} = rP + sP^2 \Rightarrow \frac{dP(t)}{dt} = rP(t)\big(1 - sN\big) \; = \; rP(t)\left(1 - \frac{N}{K}\right) ,$$

if I have copied it correctly. But first, the 'r' is not factored correctly, the rightmost term should be '$(1{+}s/r)$'. Right? Then with $K = -r/s$, the rightmost form can result. Just after that you write that $K - 1/s$, which doesn't follow in the equation. That is also in Table 1. I think it's very important to check this carefully and get it right. Oh, one other thing, why is N used in the equation? Aren't you changing that to $P(t)$?

In Equation 4, it shows $Cexp$, which should be $C\,\mathrm{exp}$, non-italicized for the 'exp'.

We noticed some misspellings in the manuscript to be corrected, for example 'Verhults' and 'Matusian.' Oh, and I noticed my name misspelled as 'Lehmann,' with two n's, in some places.

Where you estimate the crossover point as 11 ± 0.5 billion, can you really say that it can be predicted that closely, to half a billion? I would think we cannot know the distant future to predict that very accurately.

In the section on methodology, you use the term evolution in a non-biological way. That will be confusing, or at least distracting, in a paper that would be read by many biologists, as it would trigger thoughts of Darwinian evolution. A different word should be found, I would say.

In Table 1, as already mentioned, it has $K = 1/s$ in the rightmost column, which should be coordinated with the factoring discussion above. Also, it gives a population range of 1 million to 3.3 billion, but doesn't your data start at 2 million?

Below that table, it says that in Ref. 38 (our paper), 36 pairs of values (r_i, s_i) were needed for parameterization via the extended Verhulst equation. Are you saying that is what we did? I'm not thinking of it that way at all. We had only two pairs of values for r_i, s_i in the figures we are talking about—one before the discontinuity and one after. We used 44 data points, by the count I just made from our appendix, to fit a straight line and determine the first pair, r_1, s_1. And we have not thought of it as an extended Verhulst model, because it resulted from the collapse of a multi-dimensional mutualism equation to a single-dimensional equation, as explained in Figure 13 of the BioScience paper.

Related to the conclusions, I would say that we did not at all apply what Pearl and Reed did, where they used multiple re-evaluations of logistic growth (I get the feeling they did it in desperation) to try to fit what we now can represent as orthologistic growth. In orthologistic growth, as occurs in strong mutualisms, and as human populations experienced during our developing mutualism with our plant and animal agricultural partners, any carrying capacities are so far away as not to even be included in the equations. This is a strange property of the mutualism equations, which has caused misunderstandings since Volterra's time. (Volterra, however, did not seem to misunderstand them.) There is some further discussion in our e-textbook, available free at `https://conservancy.umn.edu/items/9be26a7b-cd23-4f4c-baf4-32ea64285b69` . But this whole topic perhaps deserves a separate paper discussing it further.

4. Conclusions

Thank you for thinking this through in a very different way, and I hope these comments are useful. We can remain in continuing contact if that would be helpful. And best wishes for getting your manuscript ready to publish!

Sincerely,

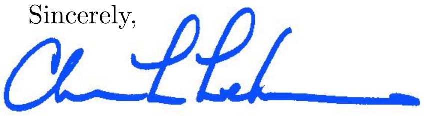

Clarence L. Lehman

Email: lehman@umn.edu
Phone: 612-325-0745 (mobile)
Office: 100C Ecology Building
Web: https://cbs.umn.edu/directory/clarence-lehman

University of Minnesota
Department of Ecology, Evolution, and Behavior, Adjunct Faculty
Morse-Alumni, Distinguished Teaching Professor
Institute on the Environment, Resident Fellow
