# Supplementary material for: Verhulst-type equation and the universal pattern for global population growth
Source: PLoS One. 2025 May 19;20(5):e0323165. doi: 10.1371/journal.pone.0323165 (PMC12088597; doi:10.1371/journal.pone.0323165)
Supplement: Attachment — Submitted filename: 2nd Response Letter 29 10 2024.pdf [file pone.0323165.s007.pdf]

Dear Editor

Attached please find the second revision of the report ‘ *Verhulst-type Equation and the Universal Pattern for Global Population Growth*‘. Please note that we decided to make a subtle change in the title, motivated by reviewers' comments. The basic title was ‘ *Verhulst Equation and the Universal Pattern for Global Population Growth* ‘

We are grateful for the reviewers' comments, which notably influenced the report. We are particularly grateful to Prof. Lehman, the author of the extended Verhulst-type equation, who is the key focus of the report.

We are happy that all reviewers see the cognitive potential of the report.

Regarding opinions of Reviewers #1 and #2

1. **Reviewers comment:** ‘ *The primary concerns centre around the originality and contribution of the work. Specifically, the reviewers note that the application of the Verhulst model is not novel, as it has been utilized frequently in prior research* ‘
THE ANSWER: the ‘deep reference’ of the report is the Verhulst equation, but this report is basically related to its qualitative extension by Lehman et al. [*PNAS* 2021; 118: e2024150118], where the successful global population in the last 12 millennia and the new concept of the per capita population growth was developed.
To stress more this point we decided to a ‘subtle’ change in the title (see above) and add the following sentence in **lines (116 - 122)**: The primary inspiration for this report was the recent report by Lehman et al. [62], which combines the mentioned basic 1st and 2nd cognitive paths for global population studies and considers the Verhulst-type scaling equation associated with the concept proposed by Pearl and Reed [85,86] and further developed by Volterra [87] and Cohen [88]. We stress this issue because such an approach essentially extends the basic Verhulst (including Two-Mode Logistic (TML) approach), often questioned for its suitability for predictive purposes regarding human populations.’
2. **Reviewers comment:** ‘*For the paper to make a more substantial contribution, the authors need to articulate their unique insights and contributions clearly. Additionally, while the authors use the TMLS (Two-Mode Logistic System) for modeling* ‘
THE ANSWER: This point is partially explained by the above comment (line 116-122). The key novelty is the introduction of the new ‘bottom–up’ approach, namely (i) numerical filtering of empirical data, (ii) their distortions sensitive analysis exploring the new analytic extension of per-capita relative population growth rate Gp, (iii) the ultimate validation test of the given scaling relations as well identification of domain in which it can be applied. This is the new path for any population data analysis, including global one.
This issue is finally stressed also in conclusions. Prof. Lehman noted this novelty and the possibility of using the proposed methodology beyond global population analysis, for instance, in biology.
3. **Reviewers comment:** ‘*one reviewer questions its suitability for predictive purposes and suggests a comparison with other models, such as ARMA and ARIMA, to validate the results*‘
THE ANSWER: **This issue is – in detail – explained in lines 95 – 116**, with the support of a new reference. Particularly it is indicated that although ARMA/ARIMA approaches are

broadly used in business, medicine, or for describing (time-limited!) development or urban centers. Reports regarding global population growth are minimal – because they cannot lead to conclusive results, particularly when considering extreme periods, as in the given report or the paper by Lehman et. al. [PNAS, 2021].

Moreover, introducing such an analysis – despite its fundamental problems – would break the consistency of the work, introducing a qualitatively different problem. It would also significantly increase the size of the report, contrary to the editor's suggestion.

4. **General suggestion:** '*Moreover, the reviewers find the theoretical section of the paper too lengthy, recommending that it be streamlined to include only the essential equations*'.
   THE ANSWER: Note that now the report contains only 10 equations, with re-arrangement not-loosing interpretations. Also some comments have been removed.
   However, please note that this requirement does not agree with points (1 – 3) above, particularly related to ARMA/ARIMA concept which had to increase the size of the report and the number of references.

Prof. Lehman comments:

Prof. Lehman was very satisfied with the given report and developed a new path concept regarding the extended Verhulst-type modeling in his recent report (PNAS, 2021). He suggested some corrections regarding equations, making them more informative and clear. It has been done. He also suggested some stylistic comments, for instance, minimizing the usage of the word 'evolution', which has a different meaning for readers from biology – related communities. Following comments of Prof. Lehman, the following sentence terminates the report: (lines 528-532) ' Finally, the authors stress that the approach proposed in the report and ref. [84] can be implemented for arbitrary time-evolving data, from biology and medicine to economic issues. The particular efficiency of such a bottom-up approach matched with the distortions-sensitive analysis can appear when local distortions, also aperiodic, distort or even hide the leading trend.'

In conclusion, we explicitly and positively considered all reviewer comments. We are happy that the cognitive value of the report emerges. Particularly, we are happy and grateful for the opinion of Prof. Lehman, the world-key researcher in population studies nowadays.

With best regards

Agata Angelika Sojecka (Univ. Econom., Katowice, Poland).
Aleksandra Drozd-Rzoska (IHPP PAS, Warsaw, Poland)
