# Supplementary material for: Verhulst-type equation and the universal pattern for global population growth
Source: PLoS One. 2025 May 19;20(5):e0323165. doi: 10.1371/journal.pone.0323165 (PMC12088597; doi:10.1371/journal.pone.0323165)
Supplement: Attachment — Submitted filename: PONE-Review2-byCL.pdf [file pone.0323165.s006.pdf]

UNIVERSITY OF MINNESOTA

Twin Cities Campus

Department of Ecology, Evolution and Behavior

College of Biological Sciences

100 Ecology Building
1987 Upper Buford Circle
St. Paul, MN 55108-6097

Office: 612-625-5700
Fax: 612-624-6777
http://www.cbs.umn.edu/eeb

MEMO TO: Agata Angelika Sojecka and Aleksandra Drozd-Rzoska
FROM: Clarence Lehman, also representing co-authors Shelby Loberg and Michael Wilson
SUBJECT: Thoughts on your revised manuscript
DATE: November 24, 2024

I just read the revision of your paper sent to me by the PlosOne editors and it looks like you've done some serious work. Getting right into it, here are some comments, line by line:

Line 18. Uses BC here and CE elsewhere. Shouldn't this be unified, such as BCE? I also noticed it later on line 130 and elsewhere.

Line 22. The difference in dates is not an important issue, but I'm wondering if yours comes up with 1968 versus our 1962 for the transition year because the smoothing that you use takes a little while to turn around? Something to think about perhaps.

Line 23. I'm not sure what the phrase "unique (universal?) reference pattern" means. The linearity of these curves is just an approximation, something of a surprising approximation it was to me. The increased population growth following World War II shows up as one of the non-linearities, and of course the major diseases and wars show up as reduced population growth nonlinearities. The generalized population growth model for a single species, or for a group of strongly mutualistic species, would be $(1/N)(dN/dt) = r + sN + s_2N^2 + \ldots + s_iN^i + \ldots$, as documented by Hutchinson, but just the linear terms approximate us and our domestic mutualists rather well. It also works in various animal populations, going back as far as Allee's *Tribolium* flour beetles.

Line 26. It says, "a single pair of growth rate and system resources (carrying capacity) coefficient patterns $(r, s)\ldots$" This makes it sound like s is the carrying capacity. The carrying capacity is actually $-r/s$, if s is negative, slowing population growth as the population gets larger. And there is no carrying capacity in the equations if s is positive, which increases the rate of population growth as the population gets larger. (By the way, the reason we named it *orthologistic* growth is because the asymptote that the population heads toward is vertical when s is positive, whereas it is horizontal at the level of the carrying capacity when s is negative, as in the formulation by Verhulst. In other words, the two asymptotes are orthogonal, hence the term orthologistic.

Line 26 again. It's just a comment about terminology. In ecology, the parameter r has long then called "the intrinsic rate of natural increase," but I didn't have a good terminology for s. Then last week when explaining this to students one more time, I thought it might be called the "speeding or slowing" parameter, depending on whether s is positive or negative. Both of those words begin with "s". I'm not saying you should use it, but I bring it up in case you want to introduce that terminology in your paper. Then I can cite your paper when I use the terms. :)

Line 28. I see you have the term "evolution" there still, which I do believe will cause confusion among biologists, because it suggests the biological evolution of a population, which continually proceeds through time.

Line 34. Can you really assure that the linearized transformation "yields optimal values of the relevant parameters?" It's certainly yields workable values, but when it smooth out the various plagues, wars, famines, and also baby booms, can we say that the parameters are "optimal?" In all of these methods of fitting the data, the parameters remain approximate.

SECOND REVIEW OF MANUSCRIPT

Line 59. I go over some of the things you are mentioning in my ecology classes, but the examples can actually start much earlier—for example with the first stone tools, that could carve hard roots that our ancestors' teeth couldn't readily chew, but could also slice someone's jugular vein, and with fire, which opened a whole new realm for food but also could burn down entire villages. I think it is interesting to talk about such challenges, as you are doing.

Line 96. The term "optimal solution" arises again. And to make it less controversial, and still accurate, you might consider saying something like "can form an excellent solution." Or if you want readers not to think you are marketing an idea, you could say "worthy solution," or even more modestly, as I mentioned earlier, "workable solution." If you seem to be pushing an idea, rather than just presenting it for evaluation, that can make some readers more skeptical, I think.

Line 129. I notice you have the population growth the way we write it in our papers, and also in our textbook, in the form of $(1/P)(dP/dt)$, but earlier as $\big(dP(t)/P(t)\big)/dt$, on line 21. Of course they're equivalent, but when I saw your alternative form, I was wondering if that might be easier for students to understand. I will have to try it both ways and see. I don't know if there's anything here that should be changed, but I just thought I would bring this up.

Line 138. Related to a comment from my previous review, not everyone agrees on what should be called the Anthropocene, so perhaps you should say something like "extending from the time that we are considering the onset of the Anthropocene."

Line 163. Just a little point, why not write it as $P(t) = P_0 e^{rt}$? Isn't that a more standard mathematical form, and also avoids the problem of whether the 'exp' should be italicized or not?

Line 175. I think you mean $K = -r/s$, with a minus sign before the quotient. Otherwise, with a positive r, the right-hand term in Equation 2 will run away and poke through infinity along a vertical asymptote, orthologistically, rather than leveling off along a horizontal asymptote as needed for logistic growth.

Line 188. There is a well-developed ecological theory formulated over the past fifty years or so by David Tilman and others that could be examined and mentioned here. It concerns limited resources interacting with population growth and leads to carrying capacities determined by resource needs. This theory was first previewed by Volterra.

Line 192. I noticed you mention Easter Island as an example. I have recently read that populations there did not proceed as thought, but I haven't looked into it at all. I noticed you don't cite a reference there. It could be something to look into more carefully.

Line 194 and forward. I have not tried to think about the historical topics you are mentioning here. It's a little beyond my range of knowledge. Some other reviewer would be better for that.

Line 249. This idea that human population growth follows a logistic equation with carrying capacities continually increasing, to allow the growth curve of $1/N\,dN/dt$ versus N to be a line of positive slope, seems to me to be an attempt by modellers of human population during the 20th century to work with a single-species equation. In fact, once agriculture started, the system must really be modelled as a multi-dimensional ecological equation that includes both humans and our agricultural mutualists. That multi-dimensional equation then collapses to a single-dimensional equation with no carrying capacity that can be computed from the parameters in the multi-dimensional model. It depends on other things completely. I did the mathematics to check and it is possible to make a one-dimensional equation with the carrying capacity increasing so rapidly that it essentially turns into an orthologistic equation. Of course there always is a carrying capacity, determined ultimately by the area of the earth for example, but that carrying capacity cannot be determined—requiring knowledge of unpredictable future social, technological, and scientific discoveries. The complete discussion of this would take quite a while. Perhaps this represents two fundamentally different concepts of population modelling. So I'll leave it at that for now. If you'd like to communicate more about this by email, or perhaps by Zoom, I would be happy to explore this further. I think it is a fundamentally important topic to work through.

SECOND REVIEW OF MANUSCRIPT

Line 258 through 265. in reviewing our paper and your explanation about it, may I suggest the following somewhat modified wording: "Very recently, Lehman et al. [62] have developed these concepts by considering global population growth in terms of three successive bio-ecological levels: (1) interactions with predators, (2) interactions with prey, and (3) intraspecific interactions. Global population changes at each level are governed by level-dependent ecological coefficients (r_i, s_i), $i = 1, 2, 3$. These led to population discontinuities progressively separating (i) a primordial phase, where pre-human ancestors interacted with their environment as other animals do, (ii) a mastery of tools, fire, and specialization phase, (iii) an agricultural phase, and finally (iv) a present controlled-fertility phase. Parameters for population growth changed at each discontinuity. These were implemented for ..." I would say "ecological coefficients" rather than "Verhulst coefficients" because I don't think Verhulst ever considered three-dimensional population growth equations, did he? So it seems misleading to say "Verhulst coefficients". Anyway, feel free to use the wording above without any acknowledgement to me—as I've written it above or with any clarifying modifications you see fit. I'm just trying to help you make your paper as clear and accurate as possible.

Line 264. Included in the above, you have "files" in "tools, files, and specialization" It should be "fire."

Line 266. I haven't checked Equation 4 completely, running out of time to finish this review.

Line 304 through 394. Again, I haven't checked the historical discussion or conclusions here. I did check the figures though. Also, I see an AD in there, which should be unified to match the other CE etc.

Line 400. I haven't checked the parameters in the table in detail, but the r parameter you list for the first domain seems plausible. However, there is something I should add. I said earlier that the carrying capacity is $-r/s$. I should have said that the *equilibrium* is $-r/s$, if that value is not negative. Then if s is negative and r is non-negative, $-r/s$ represents a stable carrying capacity K. But if s is positive and r is negative, then it represents an unstable Allee point A. So the rightmost column in the table should really be labelled "Equilibrium, carrying capacity K or Allee point A." And in the rightmost column for the first domain you have an Allee point, which corresponds numerically to the unstable equilibrium point at the left in Figure 2. On the other hand, in the rightmost column for the second domain, you have a carrying capacity. But the value in the table seems to be wrong. When I calculate it I get $-\big((-0.0028)/(-0.00000227)\big) = 1233.5$, which corresponds numerically to the stable equilibrium at the right in Figure 2. So I would say this table definitely needs a little more work.

Line 491. My name is misspelled here, with two n's instead of one.

Well, that's enough for this review I think. I encourage you to address these comments and I would be happy to have a look at the next revision, and indeed would be happy to discuss things with you as you are working on the next revision. As I said before, it is interesting to see that such different approaches can lead to similar conclusions—helping corroborate both approaches, and also the conclusions, in this important area that you are pursuing.

Sincerely,

Clarence L. Lehman

Email: lehman@umn.edu
Phone: 612-325-0745 (mobile)
Office: 100C Ecology Building
Web: https://cbs.umn.edu/directory/clarence-lehman

University of Minnesota
Department of Ecology, Evolution, and Behavior, Adjunct Faculty
Morse-Alumni, Distinguished Teaching Professor
Institute on the Environment, Resident Fellow
