# Supplementary material for: Verhulst-type equation and the universal pattern for global population growth
Source: PLoS One. 2025 May 19;20(5):e0323165. doi: 10.1371/journal.pone.0323165 (PMC12088597; doi:10.1371/journal.pone.0323165)
Supplement: Attachment — Submitted filename: Reviewers Response.pdf [file pone.0323165.s009.pdf]

Dear Editor,

Thank you very much for sending the next tour opinion regarding the paper ‘ *Verhulst-type Equation and the Universal Pattern for Global Population Growth* ‘ (PONE-D-24-03938R2).

One of Reviewers accept the current form of the manuscript. The next Reviewer, Prof. Clarence L. Lehman send a letter with some supplementary comments which should ne also addressed. Finally he expressed a general positive opinion on the report an related research.

I am very for these comments which really make the report better and more in-depth presenting arguments. I am grateful for these comments and opinion, because for me Prof. Lehman is an exceptional world authority in the field of population research and this opinion and the comments contained in it are of great importance to us, also for further research. Comments notably improved the report and we are very grateful.

Please note: following advices Figures have been improved, by adding description, so they are now ‘more communicative’. for instance for students. The responses to required additional referrnces, also indicated by the reviewer.

Below please find responses to comments, point-by-point.

1. **Reviewer:** ‘Line 18. Uses BC here and CE elsewhere. Shouldn't this be unified, such as BCE? I also noticed it later on line 130 and elsewhere.’

Response: It has been dome. In the report only CE and BCE is now used.

2. **Reviewer:** ‘Line 22. The difference in dates is not an important issue, but I'm wondering if yours comes up with 1968 versus our 1962 for the transition year because the smoothing that you use takes a little while to turn around? Something to think about perhaps.’

Response: *in line22 (Abstract), the ‘approximate’ sign has been introduced. In lines 132-133 the year 1962 instead 1968 has been corrected – since it recalls ref. [64], recalled in the above comment.* *<u>This issue is now explained via the introduction of the error+comments, Lines 394-396:</u>*
‘‘Regarding the crossover year (1968), notable is the error related to three standard deviations and the intersection of two line in Fig.3.’

3. **Reviewer:** ‘Line 23. I'm not sure what the phrase ¥unique (universal?) reference pattern" means. The linearity of these curves is just an approximation, something of a surprising approximation it was to me. The increased population growth following World War II shows up as one of the non-linearities, and of course the major diseases and wars show up as reduced population growth nonlinearities. The generalized population growth model for a single species, or for a group of strongly mutualistic species, would be (1=*N*) (*dN/dt*) = *r*+*sN* + *s*2*N*2 + *: : :* + *siNi* + *: : : ;* as documented by Hutchinson, but just the linear terms

approximate us and our domestic mutualists rather well. It also works in various animal populations, going back as far as Allee's *Tribolium* our beetles.'

Response: the words 'unique (universal)' in line 23 has been removed.

Nevertheless, (for future research) the meaning of a simple patter for the global population revealed in ref.[64] and discussed in the given report remains. For me the question arises does it can be a reference for any 'correct' scaling equation for the global population growth, which explains the word 'universal'. But this is the task for the further research and I am grateful for the above comment since it can be a significant indication.

4. **Reviewer:** Line 26: 'It says, ¥a single pair of growth rate and system resources (carrying capacity) coefficient patterns$(r; s): : :$" This makes it sound like s is the carrying capacity. The carrying capacity is actually r/s, if s is negative, slowing population growth as the population gets larger. And there is no carrying capacity in the equations if s is positive, which increases the rate of population growth as the population gets larger. (By the way, the reason we named it *orthologistic* growth is because the asymptote that the population heads toward is vertical when s is positive, whereas it is horizontal at the level of the carrying capacity when s is negative, as in the formulation by Verhulst. In other words, the two asymptotes are orthogonal, hence the term orthologistic.'

Response: *to avoid confusion the name (carrying capacity) has been removed, and only 'resources' remain', which correlates with Verhulst basic focused on foods.*

The comment is perfect, and once more important for further research, which should follow a strict reasoning pattern.

5. **Reviewer:** 'Line 28: I see you have the term ¥evolution" there still, which I do believe will cause confusion among biologists, because it suggests the biological evolution of a population, which continually proceeds through time.'

Response: *The problem is the meaning and usage of the word 'evolution' in different 'societies' of researchers. The authors of this report are associated with complex systems physics and socio-economics 'societies'. To avoid confusion for biologist we changes in lines 28 and 30 the word 'evolution; to 'changes', and a similar correction we made further in other places of the report, if it was possible/*

However, please note the title of ref. [99], which shows that using the name 'evolution' for complex systems researchers focused on the Global Population is not atypical.

6. **Reviewer:** 'Line 34: Can you really assure that the linearized transformation ¥yields optimal values of the relevant parameters?" It's certainly yields workable values, but when it smooth out the various plagues, wars, famines, and also baby booms, can we say that the parameters are ¥optimal?" In all of these methods of fitting the data, the parameters remain approximate.'

Response: *<u>Yes, I am sure that the linearized transformation as presented in the Abstract works in this way</u>. It is important that it shows a linear domain in the plot enabling the simple linear*

regression analysis yield optimal values of parameters with well defined errors. This is not possible for nonlinear routines used for scaling equations, which often has a 'flat' minima around obtained values of minima. The situation is even worst if multi-parameter impacts are considered because the problem of their 'weightings' appear.

In the statement in line 34, only the reference to explicit 'mathematic' background for P(t) data is considered, which should be the basic reference for any other consideration. This issues in/was discussed in the report. See also ref. [114] in Progress in Materials Sciences, the high impact factor journal were publication requires 6 positive reviewers opinions. It shows the unique validation possibilities of the linearized distortions sensitive approach (not for population data but for material engineering, in the given case)/. For more bio & population approach see the recent report [A.A. Sojecka, A. Drozd-Rzoska, S.J. Rzoska, *Food preservation in the Industrial Revolution Epoch: Innovative High Pressure Processing (HPP, HPT) for the 21st-Century Sustainable Society*, Foods 13, **2024**, 3028].

7. **Reviewer:** 'Line 59. I go over some of the things you are mentioning in my ecology classes, but the examples can actually start much earlier |for example with the first stone tools, that could carve hard roots that our ancestors' teeth couldn't readily chew, but could also slice someone's jugular vein, and with _re, which opened a whole new realm for food but also could burn down entire villages. I think it is interesting to talk about such challenges, as you are doing. '

Response*: In this work we wanted to focus mainly on the Industrial Revolution, which transformed the world in an extraordinary way, also leading to qualitative changes in the growth pattern of the global population. The examples given in this commentary are inspiring, and may be worth developing in further work from pre-historical times through the extraordinary changes in the Neolithic and at the border of the Bronze Age, in correlation with environmental and ecological constraints. This is an issue worth further research, especially in the context of recent and surprising discoveries of Neolithic urban complexes, e.g. in present-day Turkey Republik.*

8. **Reviewer:** 'Line 96: The term ¥optimal solution" arises again. And to make it less controversial, and still accurate, you might consider saying something like ¥can form an excellent solution." Or if you want readers not to think you are marketing an idea, you could say ¥worthy solution," or even more modestly, as I mentioned earlier, workable solution." If you seem to be pushing an idea, rather than just presenting it for evaluation, that can make some readers more skeptical, I think.'

Response*: Please see the name in* Lines 95/96: 'workable solution'.

This comment is a perfect advise.

9. **Reviewer:** 'Line 129. I notice you have the population growth the way we write it in our papers, and also in our textbook, in the form of $(1/P)(dP/dt)$, but

earlier as $(dP(t)/P(t)/dt$, on line 21. Of course they're equivalent, but when I saw your alternative form, I was wondering if that might be easier for students to understand. I will have to try it both ways and see. I don't know if there's anything here that should be changed, but I just thought I would bring this up.'

Response: *Please note the supplemented Line 129, which shows the explicit discrete counterpart of the analytic form of* G_P *parameter. I think it can help ;less experienced' readers (*Lines 128/129*): '…* $G_P^i = (1/P_i)(\Delta P_i/\Delta t_i) = [(\Delta P_i/P_i)/\Delta t_i]$ vs. P_i, where …'

10. **Reviewer:** 'Related to a comment from my previous review, not everyone agrees on what should be called the Anthropocene, so perhaps you should say something like ¥extending from the time that we are considering the onset of the Anthropocene.'

Response: *Please see the correction in* lines 137-138*: '*This report focuses on the meaning of this exceptional pattern of G_P for global population changes in the period extending from the time that we are considering the onset of the Anthropocene.'

11. Reviewer: 'Line 163. Just a little point, why not write it as $P(t)$ = P0ert? Isn't that a more standard mathematical form, and also avoids the problem of whether the `exp' should be italicized or not?'

Response: Please see the current form of Line 163/164:

$$\frac{dP(t)}{dt} = rP(t) \quad \Rightarrow \quad P(t) = P_0e^{rt} = P_0exp(rt) \qquad (1)$$

which explains the issue for readers.

It shows the link between both forms of presentations. Nevertheless, in the subsequent part of the report the notation ' ' is used since it follow the dominant way of presentation in complex systems science and complex systems physics, and in our opinion offers a more clear insight. Following such notation a reference*: italics* for*'* $exp(rt)$ … is obligatory.

12. **Reviewer:** 'Line 175. I think you mean K = -r/s, with a minus sign before the quotient. Otherwise, with a positive r, the right-hand term in Equation 2 will run away and poke through innity along a vertical asymptote, orthologistically, rather than leveling off along a horizontal asymptote as needed for logistic growth.'

Response: it has been corrected (see line 176)

13. **Reviewer:** 'There is a well-developed ecological theory formulated over the past _fifty years or so by David Tilman and others that could be examined and mentioned here. It concerns limited resources interacting with population growth and leads to carrying capacities determined by resource needs. This theory was first previewed by Volterra.'

Response: *Please note the new sentence in* Lines 191-194:

'Worth recalling is the model developed by Tilman [94,95] and followers [96], which discussed resources interacting with population growth, which indicates carrying capacities determined by resource needs.

It was associated with 3 new references [94,95,96]. '

14. Reviewer: 'Line 192I noticed you mention Easter Island as an example. I have recently read that populations there did not proceed as thought, but I haven't looked into it at all. I noticed you don't cite a reference there. It could be something to look into more carefully.'

Response: *Please note supplementations answering this comment, in* Lines 195-201:

'Recently, the Verhulst-type pattern has been shown for human population changes on Easter Island (Rapa Nui), the Pacific island, located well remote from other islands and the South America mainland [94]. It is worth mentioning that recent studies have shown that this previously dominant picture, linking ecological constraints and population changes, was substantially changed by research showing the devastating impact of contact with European sailors and later marauders who enslaved people on the South American mainland [97,99].

It was associated with 2 new references [97,99].

15. Reviewer: 'Line 194 and forward. I have not tried to think about the historical topics you are mentioning here. It's a little beyond my range of knowledge. Some other reviewer would be better for that.'

Response: *The above comment, related to (P.14) response this question/problems – particularly when taking into account added references from Nature and Science Advances (both 2024), written in a nice, even popular, way.*

15. Reviewer: Line 249. This idea that human population growth follows a logistic equation with carrying capacities con-tinually increasing, to allow the growth curve of 1=*N dN/dt* versus *N* to be a line of positive slope, seems tome to be an attempt by modellers of human population during the 20th century to work with a single-species ……..If you'd like to communicate more about this by email, or perhaps by Zoom, I would be happy to explore this further. I think it is a fundamentally
important topic to work through.'

Response: *It is a a very interesting comment, not related to the supplementation in the report but to opinions exchanges and discussions. We are very happy to this, and we will contact as soon as the given paper appears. Earlier could be a bit unfair for reviewing rules.*

15. Reviewer: 'Line 258 through 265. in reviewing our paper and your explanation about it, may I suggest the following somewhat modi_ed wording: ¥Very recently, Lehman et al. [62] have developed these concepts by considering global population growth in terms of three successive bio-ecological levels: (1) interactions with predators, (2) interactions with prey, and (3) intraspeci_c interactions. Global population changes at each level are governed by level-dependent ecological coefficients (*ri; si*), *i*=1*;* 2*;* 3. These led to population discontinuities progressively separating (i) a primordial phase, where pre-human ancestors interacted with their environment as other animals do, (ii) a mastery of tools, fire, and specialization phase, (iii) an agricultural phase,

andfinally (iv) a present controlled-fertility phase. Parameters for population growth changed at each discontinuity. These were implemented for :::″ I would say ¥ecological coefficients″ rather than ¥Verhulst coefficients″ …

Response ‘ *Exactly the above description of the (fantastic!) ref. [62] has been including in the report.* They are Lines 268-276 in the current version of the reports.

<u>This resume is really better. Thank You.</u>

15. Reviewer: ‘Line 264. Included in the above, you have ¥_les″ in ¥tools, _les, and specialization″ It should be fire.″’

Response: It was a misprint. It has been corrected.

16. **Reviewer:** ‘Line 304 through 394. Again, I haven't checked the historical discussion or conclusions here. I did check the Figures though. Also, I see an AD in there..,’

Response: *We are sure for the (very soft) historical discussion. Although we do not specialize in history, this branch of knowledge is more than a hobby for us.*

We went through the paper and now only CE and BCE appears.

17. **Reviewer:** ‘Line 400. I haven't checked the parameters in the table in detail,…. So I would say this table definitely needs a little more work’

Response: please see the corrected Table and the discussion supplement in lines*: . Note that values of coefficients are directly taken from the linear regression fit, including the error. We stress in the text that their substitution to the Verhulst equation do nit yield P(t) portrayal in the 1st or the 2nd period. In cam mean that the derivative equation leading to the given plot GP(P) vs. P offers a nice general description for changes, but the Verhulst equation not (if we use single (which can mean ;effective) sets of parameters for each domain, It may indicate that another scaling equation, for which a similar differential equation (approximately) can be derived should be the target in searching for the global population description.*

18. **Reviewer:** ‘Line 491. My name is misspelled here, with two n's instead of one.′

Response: Sorry for the misprint. It has been corrected.
