# Supplementary material for: Verhulst-type equation and the universal pattern for global population growth
Source: PLoS One. 2025 May 19;20(5):e0323165. doi: 10.1371/journal.pone.0323165 (PMC12088597; doi:10.1371/journal.pone.0323165)
Supplement: Attachment — Submitted filename: PLOS-Review3-CL.pdf [file pone.0323165.s008.pdf]

UNIVERSITY OF MINNESOTA

Twin Cities Campus

Department of Ecology, Evolution and Behavior

College of Biological Sciences

100 Ecology Building
1987 Upper Buford Circle
St. Paul, MN 55108-6097

Office: 612-625-5700
Fax: 612-624-6777
http://www.cbs.umn.edu/eeb

MEMO TO: Agata Angelika Sojecka and Aleksandra Drozd-Rzoska
FROM: Clarence Lehman, also representing co-authors Shelby Loberg and Michael Wilson
SUBJECT: Thoughts on your revised manuscript
DATE: February 20, 2025

I have read the edits for the most recent revision of your paper sent to me by the PLOSONE editors and I see you've made a careful effort to address the issues brought up earlier. I looked over each of your changes and they looked good, subject to the editor's final judgement of course. I'll number my few new points below.

Point 1. Beyond your editing that I just checked, I think I had better check each of your displayed equations, because it is so easy to get signs inverted, subscripts mismatched, or other complications in equations. The first equation on line 163 looks fine to me.

Point 2. I'm wondering about the second equation, clipped from line 173 of the revised manuscript you sent:

173 $$\frac{dP(t)}{dt} = rP + sP^2 \;\Rightarrow\; \frac{dP(t)}{dt} = rP\left(1 + \frac{1}{K}P\right) \;\Rightarrow\; \frac{1}{P}\frac{dP(t)}{dt} = r + \frac{r}{K}P \qquad (2)$$

I'll just repeat the process step by step to check things out. First, I get

$$\frac{dP(t)}{dt} = rP + sP^2 \Rightarrow \frac{dP(t)}{dt} = rP\left(1 + \frac{s}{r}P\right)$$

That works, but now the K has to be inserted. So with $K = -r/s$, this should become

$$\frac{dP(t)}{dt} = rP\left(1 - \frac{1}{K}P\right) \Rightarrow \frac{1}{P}\frac{dP(t)}{dt} = r - \frac{r}{K}P$$

Oops, it differs in the minus sign. Which one of the above is wrong? Rather than thinking about it deeper right now, I'll just graph the two differential equations, from the second part of Equation 2 above, with a plus sign and then with a minus sign. I'll set $K = 100$, $r = 1$, and $P_0 = 1$. Here is that graph:

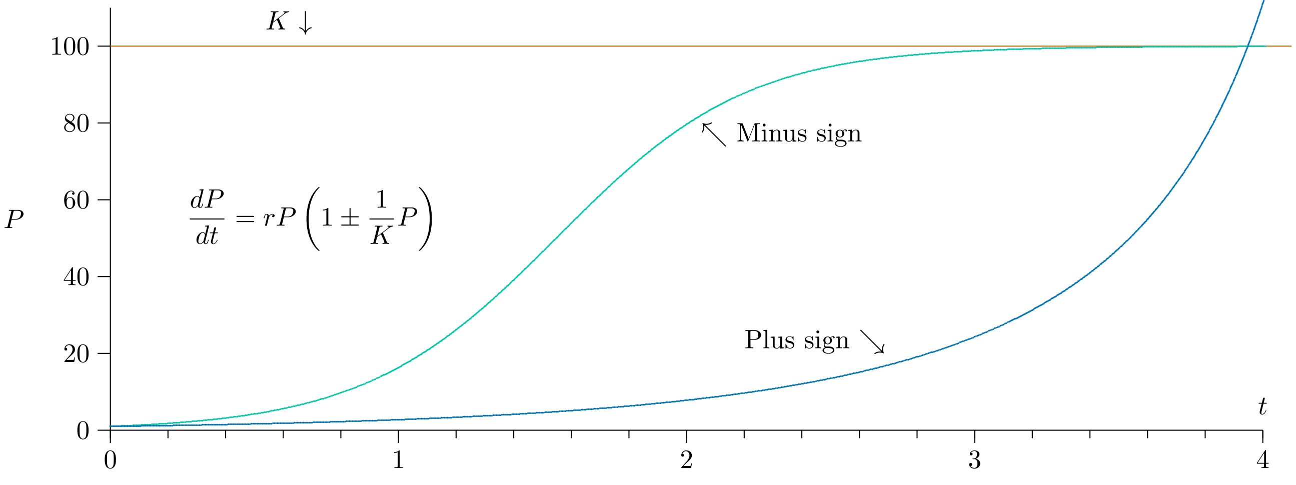

Okay, the minus sign is correct, plus sign is wrong. It looks like you'll have to change Equation 2 to use '$-\frac{1}{K}P$' and '$-\frac{r}{K}P$' in the two at right. The '$+sP^2$' in the first part is correct. You'll have to check that over carefully.

Upon reflection, now it's easy to see why. Look at the differential equation. The intrinsic growth rate is r, a positive number for a population that will grow, and K is the carrying capacity, also a positive number for

a population that will survive. But for a population to reach an equilibrium, as the K implies, something will have to slow the growth, and with only positive numbers, nothing will. Growth becomes orthologistic, as described in our paper.

After all this, I just went to Wikipedia* to see what it said about this, and down the page to Verhulst I see that it represents the logistic equation with the minus sign. I could have done that right away. But anyway, this effort was informative.

This effort also brings up what I now try to do in all my work, and that is never to trust an equation without plotting special cases of it graphically. That is pretty easy to do nowadays. Another point related, which David Tilman strongly advises me, is nowadays always to make sure that every graph in the paper can be reproduced from data supplied with the paper, or from data readily available. We did that in the PNAS paper, and also included software code to calculate the parameters we used in the paper. That allows the figures to be compared with other sources of data, extended into the future as new data become available, or adapted to other purposes.

Point 3. Your Equation 3 appears to be correct. To make an independent check, just for my own comfort, I also graphed that and it matched the green curve above perfectly. (Oh, by the way, I forgot to mention that the green curve above used $r = 3$ to get it to rise rapidly enough.)

Point 4. Finally, the last equation:

279 $$G_P = \frac{1}{P(t)}\frac{\Delta P(t)}{\Delta t} = r_i \pm s_i P(t) \Rightarrow \qquad P(t) = \sum \frac{1}{\left(P_0 \mp (s_i/r_i) e^{-r_i t} \pm (s_i/r_i)\right)} \tag{4}$$

Well, it looks like that is incorrect. First, the right-most equation has P_0 as the first term in the denominator, but isn't it supposed to be $1/P_0$? Also, representing the population at time t as a sum (Σ) isn't quite it. The system runs along with a fixed set of parameters, starting at an initial value of P_0, and when the parameters change at some time $t = t_i$, due to a change in human events—such as when mutualism with newly domesticated animals developed and changed the fundamental population dynamics—the equations simply continue with the new parameters and with a new P_0 that is equal to the final $P(t_k)$ of the previous step. So it isn't really a sum, it's really an equation with suddenly changing parameters, with what might be called externally imposed bifurcations.

Point 5. As I've said before, what we did in the PNAS paper, and earlier in the BioScience paper, doesn't seem like a "multi-parameter Verhulst-type model," but you'll have to explain it in the way that you think will be most understandable to readers in your field. Perhaps describing it as a Verhulst-type model will make connections for them. But I have to say that it isn't really a Verhulst model, that it only reduces to a Verhulst-style model in modern times, once the kind of voluntary reductions in population growth appeared so recently.

In summary, I want to offer my best wishes for getting this to its final stage, and to thank you for the hard work you have dedicated to this important topic!

Sincerely,

Clarence L. Lehman

Email: lehman@umn.edu
Phone: 612-325-0745 (mobile)
Office: 100C Ecology Building
Web: https://cbs.umn.edu/directory/clarence-lehman

University of Minnesota
Department of Ecology, Evolution, and Behavior, Adjunct Faculty
Morse-Alumni, Distinguished Teaching Professor
Institute on the Environment, Resident Fellow
