# Supplementary material for: Verhulst-type equation and the universal pattern for global population growth
Source: PLoS One. 2025 May 19;20(5):e0323165. doi: 10.1371/journal.pone.0323165 (PMC12088597; doi:10.1371/journal.pone.0323165)
Supplement: Attachment — Submitted filename: REsponse Letter 3rd.docx [file pone.0323165.s010.docx]

Dear Editor,

Attached please find the revised manuscript ‘*Verhulst-type Equation and the Universal Pattern for Global Population Growth* ‘ ref. PONE-D-24-03938R3

It is the 3rd revision of the manuscript, we hope the last revision terminates 14^th^ month lasting cognitive ‘adventure’ associated with the submission to PlosONE.

**Two reviewers supported the publication (YES). The 3^rd^ Reviewer – Prof. Lehman, suggested some additional corrections.**
We are very grateful to Prof. Lehman for his inspiring comments and suggestions. They led to a significant improvement in the report and progressed understanding and explaining puzzling issues for the given problem.

There are two essential comments of Prof. Lehman

1. The first one is related to the correctness of some equations, particularly related to the chapter ‘ ***Remarks on Malthus and Verhulst equations***‘
2. The second one is related to the general name or more – consequences – of ‘ multi-parameter Verhulst-type model ‘

**Response:**

- All equation are now re-tested. Note that the notation referred to the basic (standard) Verhulst presentation, and the reference definition Gp =r – sP is used for the whole paper.
- Please note that the chapter ***Remarks on Malthus and Verhulst equations*** has been re-written and also presents those facts in a way that can be interesting for students in following the topic step-by-step
- Professor Lehman's breakthrough ‘empirical’ finding is related to Fig. 3 in ref. [62], portrayed by lines with different slopes, which means signs of the resources s parameter. The way to the solution of this grand mystery is now commented/explained at the end of the ‘***Remarks***…..’ chapter using the Cohen approach.
- the next issue and for us grand mystery – but also undoubtful ‘emprirical’fact/ reference is the fair portrayal in a ‘multi-parameter’ Verhulst patter.

Please, note the slightly supplemented Figure 2 (with comments below the figure) showing the polynomial portrayal and the comment below.

The report has also been ‘cleaned’ in depth. We hope that this supplementation/correction finishes the 14^th^ months' lasting story associated with the submission to PlosONE. Some new questions have appeared over this long time, but they can only be the target of further studies and reports.

Agata Angelika Sojecka	and Aleksandra Drozd-Rzoska
